# Female sexual dysfunction in newly diagnosed egyptian patients with neuromyelitis optica spectrum disorder

**DOI:** 10.1186/s12883-022-02648-8

**Published:** 2022-03-24

**Authors:** Alaa ELmazny, Sara Salama, Mona Hussein, Eman Hany Elsebaie, Rehab Magdy

**Affiliations:** 1 Department of Neurology, Faculty of Medicine, Cairo University, Cairo, Egypt; 2 Internal Medicine Department, Arabian Gulf University, Manama, Bahrain; 3 Department of Neurology, Faculty of Medicine, Alexandria University, Alexandria, Egypt; 4 Department of Neurology, Faculty of Medicine, Beni-Suef University, Salah Salem Street, Beni-Suef, 62511 Egypt; 5 Public health and community medicine department, Faculty of Medicine, Cairo University, Cairo, Egypt

**Keywords:** NMOSD, Female sexual dysfunction, BDI, FSS, MMAS

## Abstract

**Background:**

Few research works have explored female sexual dysfunction (FSD) in patients with Neuromyelitis optica spectrum disorder (NMOSD) which remains an ignored disease symptom. This work aimed to describe the frequency, patterns, and predictors of FSD in a sample of newly diagnosed AQP4-ab seropositive NMOSD patients.

**Methods:**

This case-control study was conducted on 28 seropositive NMOSD patients and 31 age matched healthy controls. All included patients were asked to privately fill and hand back the following questionnaires: female sexual function index questionnaire (FSFI), Beck depression inventory II (BDI) and fatigue severity scale (FSS). Also, Modified Modified Ashworth scale (MMAS) and Expanded disability status scale (EDSS) were applied to all included patients.

**Results:**

NMOSD patients had significantly lower total FSFI scores and significantly higher BDI and FSS scores than controls (*P* < 0.001). FSS scores were negatively correlated with total scores of FSFI as well as desire, lubrication, orgasm, and satisfaction scores. BDI scores was negatively correlated with desire and orgasm scores. The uncorrected visual FS score was negatively correlated with lower total scores of FSFI as well as arousal, orgasm, and satisfaction scores. The pain score was negatively correlated with the scores of the MMAS. The only predictors of FSFI total score were fatigue and visual disability. Visual disability was also a predictor of dysfunction in arousal and satisfaction domains, whereas spasticity in the lower limbs predicted sexual related pain.

**Conclusions:**

Sexual dysfunction in patients with NMOSD is strongly related to fatigue, depression, visual disability, and lower limbs spasticity.

## Introduction

Neuromyelitis optica spectrum disorder (NMOSD) is a central nervous system autoimmune disease that preferentially targets the optic nerves and spinal cord and has a clear female preponderance, with female to male ratios ranging from 3:1 to 9:1 in aquaporin 4 antibody (AQP4-ab) seropositive patients [1].

Female sexual dysfunction (FSD), defined as reported personal distress related to sexual desire, arousal, orgasm, and/or pain, is influenced by various organic and psychological factors [2, 3]. In several demyelinating and neurodegenerative central nervous system disorders, FSD remains a common yet an under-recognized comorbidity [4]. Studies reported a prevalence of sexual dysfunction of 40–80% in women with multiple sclerosis (MS), anytime within the disease course and even in patients with minimal disability [5–8].

As opposed to MS, few studies have explored FSD in NMOSD, which remains a disregarded aspect of the disease [9, 10].

Aim of the current work was to describe the frequency, patterns, and predictors of female sexual dysfunction in a sample of newly diagnosed AQP4-ab seropositive NMOSD patients.

## Materials and methods

### Study design and participants

This case-control study was conducted on 59 sexually active female subjects. Twenty-eight seropositive NMOSD patients according to the 2015 International Panel for NMOSD criteria [11] and thirty-one healthy, literate, non-circumcised controls. The healthy controls were selected from the patients’ relatives so that the cultural level and socioeconomic status were close between the two groups. They were free from any medical illness and matched with the included patients regarding age, sex, and years of marriage. All patients were recruited at the time of their initial diagnosis from Beni-Suef University Hospital, Egypt, Neurology clinic between June 2018 and January 2021. None were on immunosuppressants at the time of enrollment.

Exclusionary criteria were illiteracy, ages < 18 and > 45 years, circumcision, disease onset > 1 year, relapses at the time or three months before participation, an EDSS score of > 6.5, endocrine disorders including diabetes mellitus, pregnancy, lactation, use of hormonal contraceptives, antidepressants, and muscle relaxants.

### Measures

**Fig. 1:**
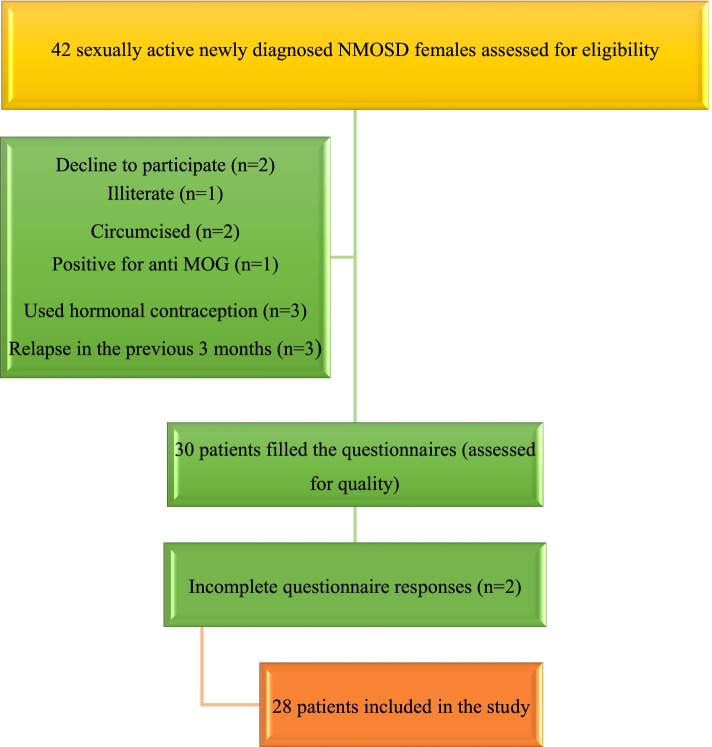
Flowchart for patients’ enrollment. The abbreviations mentioned in the figure: NMOSD: neuromyelitis optica spectrum disorder; MOG: myelin oligodendrocyte glycoprotein

All subjects were asked to privately fill and hand back the validated Arabic versions of the following: patients’ flow diagram is outlined in Fig. 1

1. Female sexual function index questionnaire (FSFI) [12]

 

A 19-item scale assessing the following six aspects of female sexual function; desire, arousal, lubrication, orgasm, satisfaction, and pain over the prior 4 weeks. The overall scale score ranges from 2 to 36 and is generated by adding all the domains’ scores. The optimal cutoff value for diagnosing FSD in the validated Arabic version used in our study is 28.1. Lower scores on the overall FSFI or any subscales indicate worse sexual function.

2) Beck depression inventory II (BDI): [13]

 

A 21-item scale that detects the presence and severity of depression. Each of the 21 items corresponds to a depression symptom. Items are rated on a 4-point intensity scale, and scores are added to give a total from 0 to 63. Higher total scores represent more severe depression symptoms.

3) The fatigue severity scale (FSS): [14]

A 9-statement scale rates fatigue severity and its impact on the patient’s activities. The possible responses range from 1 (strongly disagree) to 7 (strongly agree). The total scores are reported as the mean score of the 9 items. The higher the score, the greater the fatigue severity.

The following scales were also applied to all patients:

1. The Modified Modified Ashworth Scale (MMAS) [15]

A clinical scale is used to grade muscle spasticity and resistance to passive movements. It is a 5-point scale with a grade score of 0 to 4. A score of zero indicates no resistance and 4 indicates that the affected parts are rigid in flexion or extension.

2) Expanded disability status scale (EDSS): [16]

EDSS scores range from zero to 10 in 0.5 increments that represent higher levels of disability. Scoring is based on eight functional systems (visual, brain stem, pyramidal, sensory, cerebellar, bladder/bowel, cerebral functions, and ambulation). In addition to the total EDSS score used to assess patients’ physical functioning, the uncorrected visual and bladder functional system (FS) scores, converted to lower scores before they are reflected in the total score, were calculated separately to assess and quantify symptoms severity.

### Sampling

The sample size was calculated using G*Power version 3.1.9.7 Software based on a study conducted by Y Zhang, Q Zhang, Z Shi, H Chen, J Wang, C Yan, Q Du, Y Qiu, Z Zhao and H Zhou [9]. The probability of type I error (α) was 5%, effect size = 0.97, noncentrality parameter λ = 3.69, critical t = 2, and df = 56. A total sample size of 58 subjects was required to achieve a statistical power (1–β) 95%.

### Statistical analysis

The statistical package for social science (SPSS version 21) was used for data analysis. Simple descriptive statistics (arithmetic mean and standard deviation) were used to summarise normal quantitative data and frequencies used for qualitative data. The bivariate relationship was displayed in cross-tabulations, and a comparison of proportions was performed using the chi-square and Fisher’s exact tests where appropriate. An Independent T-test was used to compare normally distributed quantitative data. Pearson correlation was used to compare normally distributed quantitative data. The significance level was set at a probability (P) value < 0.05. Linear regression models were performed for FSS, BDI, uncorrected visual FS, MMAS, number of relapses in the first year, total EDSS and uncorrected bladder FS as dependent factors.

## Results

In the patients’ group, the age ranged from 22 to 45 with a mean age of 34.4 ± 5.3 years, whereas the ages ranged from 21 to 45 in the control group with a mean age of 33.6 ± 5.7 years. Both groups were matched regarding age and years of marriage (*P*- value 0.558 and 0.081, respectively). Clinical characteristics of the patients’ group are illustrated in Table 1. The mean value of disease duration was 8.214 (3.64) months. The uncorrected visual FS score ranged from 0 to 6, the uncorrected bladder FS score ranged from 0 to 3, and the Modified Ashworth scale ranged from 0 to 3.

**Table 1:** Clinical characteristics of NMOSD patients

Patients (*n *= 28)		
Disease duration in months [Mean (SD)]		8.214 (3.64)
Total EDSS [Median (IQR)]		5 (4-6)
Uncorrected visual FS score [Median (IQR)]		2 (1-4.75)
Uncorrected bladder FS score [Median (IQR)]		1 (0-2)
Modified Ashworth scale [Median (IQR)]		2 (0.25-3)
1st attack [n (%)]	Optic neuritis	14 (50.0%)
	Myelitis	11 (39.3%)
	Simultaneous optic neuritis and myelitis	3 (10.7%)
2nd attack [n (%)]	No	13 (46.4%)
	Optic neuritis	4 (14.3%)
	Myelitis	10 (35.7%)
	Brain stem	1 (3.6%)

*IQR* interquartile range, *EDSS* Expanded disability status scale, incorrected visual \ bladder FS score: Functional System Score

### Sexual functions, fatigue, and depression scales in NMOSD patients compared to controls

**Fig. 2:**
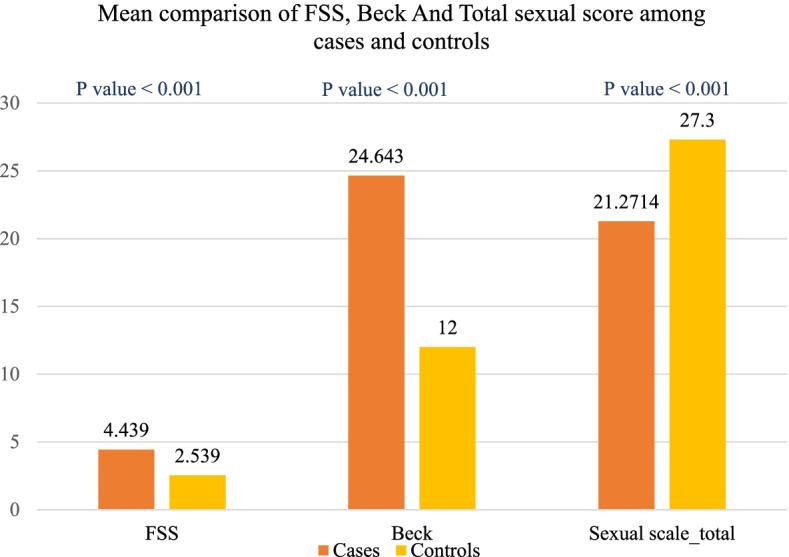
Sexual functions, fatigue, and depression scales in NMOSD patients compared to controls. FSS: Fatigue severity scale

The total scores of BDI and FSS were significantly higher among NMOSD patients compared to controls. Also, the total FSFI score was significantly lower in patients as opposed to controls (Fig. 2). All NMOSD patients included in the study had sexual dysfunction (scored ≤ 28.1 on the total score of FSFI). For all FSFI domains, patients had significantly lower scores compared to controls except for lubrication and orgasm (Table 2).

**Table 2:** Scores of different female sexual domains in patients versus controls

	Patients (*n *= 28)	Controls (*n *= 31)	*P*-value
FSFI [Mean ± SD]			
Total score	21.2 ± 4.3	27.3 ± 3.6	< 0.001*
Desire	2.9 ± 1.2	3.7 ± 1.02	0.018*
Arousal	3.2 ± 1.1	3.9 ± 0.9	0.005*
Lubrication	4.6 ± 0.88	4.5 ± 0.5	0.577
Orgasm	3.8 ± 1.2	4.1 ± 0.6	0.249
Satisfaction	3.6 ± 1.4	4.7 ± 0.4	< 0.001*
Pain	3.1 ± 0.7	6.5 ± 1.1	< 0.001*

*FSFI* Female sexual function index questionnaire, *SD* standard deviation

*P-value < 0.05 is considered significant

### Female sexual dysfunction in relation to different clinical parameters

Higher fatigue level (assessed by FSS) was associated with lower total scores of FSFI as well as desire, lubrication, orgasm, and satisfaction scores. On the other hand, higher BDI scores were associated only with lower desire and orgasm scores (Table 3).

**Table 3:** Correlations between scores of FSFI and scores of different clinical scales

		FSS	BDI	EDSS	uncorrected bladder FS score	uncorrected visual FS score
FSFI- Total score	*r*	-0.608	-0.365	-0.141	-0.144	-0.701
	*P*	0.001*	0.056	0.473	0.466	<0.001*
Desire	*r*	-0.524	-0.528	-0.074	-0.200	-0.344
	*P*	0.004*	0.004*	0.708	0.307	0.073
Arousal	*r*	-0.351	-0.047	0.027	0.071	-0.546
	*P*	0.067	0.813	0.891	0.721	0.003*
Lubrication	*r*	-0.382	-0.219	-0.341	-0.348	-0.364
	*P*	0.045*	0.264	0.075	0.069	0.057
Orgasm	*r*	-0.500	-0.402	0.063	0.109	-0.541
	*P*	0.007*	0.034*	0.752	0.580	0.003*
Satisfaction	*r*	-0.388	-0.300	-0.083	-0.058	-0.687
	*P*	0.041*	0.121	0.674	0.768	<0.001*
Pain	*r*	-0.154	0.310	-0.283	-0.265	-0.106
	*P*	0.433	0.108	0.144	0.173	0.591

*FSFI* Female sexual function index questionnaire, *BDI* Beck depression inventory, *FSS* fatigue severity scale, *EDSS* Expanded disability status scale, incorrected visual \ bladder FS score: Functional System Score

*P-value < 0.05 is considered significant

The uncorrected visual FS score was associated with lower total scores of FSFI as well as arousal, orgasm, and satisfaction scores. On the other hand, there was no significant correlation between total EDSS or the uncorrected bladder FS score and any sexual domains (Table 3).

Regarding the pain domain, the pain scores were negatively correlated with the number of relapses during the 1st year (*r* = -0.605, *P* = 0.001) as well as scores of the Modified Modified Ashworth scale (*r* = -0.756, *P* = < 0.001).

### Predictors of female sexual dysfunction among NMOSD patients

Using a linear regression analysis model, the only predictors of FSFI-Total score were fatigue and visual disability.

Visual disability was also a predictor of dysfunction in arousal and satisfaction domains, whereas spasticity in the lower limbs predicted sexually related pain (Table 4).

**Table 4:** Predictors of female sexual dysfunction among patients by linear regression analysis

Models		B	Odds ratio	*P*-value	95.0% CI	
					Lower	Upper
FSFI-total score^a^	Uncorrected visual FS score	-1.219	-0.560	**<0.001**	-1.784	-0.654
	Fatigue severity scale	-1.287	-0.419	**0.003**	-2.084	-0.489
	Constant	29.770		<0.001		
Desire score^a^	Fatigue severity scale	-0.299	-0.341	0.082	-0.639	0.041
	Beck depression inventory	-0.039	-0.349	0.075	-0.082	0.004
	Constant	5.238		<0.001	3.870	6.607
Arousal score^a^	Uncorrected visual FS score	-0.293	-0.546	**0.003**	-0.475	-0.112
	Constant	3.831		<0.001	3.285	4.377
Satisfaction score^a^	Uncorrected visual FS score	-0.447	-0.628	**<0.001**	-0.667	-0.227
	Fatigue severity scale	-0.178	-0.177	0.249	-0.488	0.132
	Constant	5.397		<0.001	4.036	6.758
Pain score^a^	N of relapses in the 1st year	-0.388	-0.267	0.084	-0.832	0.056
	Modified Ashworth scale	-0.385	-0.608	**<0.001**	-0.579	-0.192
	Constant	4.344		<0.001	3.744	4.943

*FSFI* Female sexual function index questionnaire, *EDSS* Expanded disability status scale, *FS score* Functional System Score, *CI* confidence interval

*P*-value < 0.05 is considered significant

^a^ Dependent variables

## Discussion

Sexual functioning is not routinely addressed by health professionals in many Middle Eastern countries because of the general culture of conservatism and modesty when discussing sexual matters. Despite the recognized importance of sexual problems in many neurological disorders, this aspect of illness has been poorly estimated in clinical practice. It remains underreported, underdiagnosed and undertreated, affecting not only the patients’ quality of life but their partners’ as well.

To date, few studies addressed FSD in NMOSD [9, 10], and none to our knowledge was conducted in the Middle East and North Africa region or newly diagnosed patients.

The results of our study confirmed the findings previously reported by Y Zhang, Q Zhang, Z Shi, H Chen, J Wang, C Yan, Q Du, Y Qiu, Z Zhao and H Zhou [9]. Sexual dysfunction (SD) was significantly higher in NMOSD patients than in healthy controls. However, SD was observed in 100% of our patients versus 45.3% of their female patients. This difference might be attributed to our smaller sample size, different patients’ aquaporin 4 status and higher FSIS scale cut off value in the Arabic version we used (28.1) compared to the Chinese version used in their study (23.45).

Fatigue and depression are prevalent disabling NMOSD symptoms. In NMOSD, fatigue could occur as a symptom of the disease itself and further amplified by depressive episodes [17, 18].

Significantly, higher fatigue and depression scale scores were recorded in our patients compared to their healthy counterparts.

Sexual dysfunction has direct contributions from depression and fatigue, and both could be noted as modifiable factors that may represent potential targets for SD prevention and treatment.

Our results showed that fatigue correlated with overall Sexual functions and the desire, orgasm, and satisfaction domains. In contrast, higher BDI scores were associated with lower desire and orgasm scores.

Good perception of external stimuli is critical for sexual desire, arousal, and satisfaction [19–21]. Previous studies have shown that vision, olfaction, hearing, taste, or touch deficits impair sexual functioning of varying degrees [22–24]. Patients with visual impairment may not perceive visible sexual cues resulting in bringing on individuals’ worsening sexual functioning. In the current study, Visual disability was a predictor of the overall and domain-specific sexual functions (arousal and satisfaction).

Pelvic floor hypertonic disorders are conditions that occur concurrently with muscular hypertonia or spasticity [25, 26]. Pelvic floor related sexual dysfunction comprises dyspareunia, vaginismus, and pelvic pain [27]. This might explain why lower limb spasticity is predicted and correlated with sexual pain in our patients.

Sexual function recovery is no less important than any other aspect of rehabilitation in NMOSD. Predictors of SD outlined in this study can guide health care practitioners towards proactive practice to provide proper evaluation and counselling to patients at risk.

Several limitations of the current study exist. The small number of patients, the single centre design, and the lack of supporting imaging data. Also, we didn’t exclude patients on pregabalin. Furthermore, the non-inclusion of the sensory FS score in our assessment was considered another limitation.

## Conclusions

Sexual function assessment is critical in NMOSD patients, even if they were newly diagnosed. Fatigue, depression, visual disability, and lower limbs spasticity should promote screening for the relevant SD domains reviewed in our study. Improving the abovementioned contributors may represent possible targets for FSD treatment in such patients.

## Data Availability

The datasets generated and/or analyzed during the current study are not publicly available (out of respect of patients privacy), but are available from the corresponding author on reasonable request.

## References

[1] Jarius S, Ruprecht K, Wildemann B, Kuempfel T, Ringelstein M, Geis C, Kleiter I, Kleinschnitz C, Berthele A, Brettschneider J (2012). Contrasting disease patterns in seropositive and seronegative neuromyelitis optica: A multicentre study of 175 patients. J Neuroinflammation.

[2] Raina R, Pahlajani G, Khan S, Gupta S, Agarwal A, Zippe CD (2007). Female sexual dysfunction: classification, pathophysiology, and management. Fertility Sterility.

[3] Barton D, Joubert L (2000). Psychosocial aspects of sexual disorders. Aust Fam Physician.

[4] Mazzariol C, Di Tonno F, Piazza N, Pianon C (2010). [Sexual dysfunctions in female with neurological disorders]. Urologia.

[5] Tzortzis V, Skriapas K, Hadjigeorgiou G, Mitsogiannis I, Aggelakis K, Gravas S, Poulakis V, Melekos MD (2008). Sexual dysfunction in newly diagnosed multiple sclerosis women. Multiple Sclerosis J.

[6] Lew-Starowicz M, Rola R (2013). Prevalence of Sexual Dysfunctions Among Women with Multiple Sclerosis. Sex Disability.

[7] Guo ZN, He SY, Zhang HL, Wu J, Yang Y (2012). Multiple sclerosis and sexual dysfunction. Asian J Androl.

[8] Zorzon M, Zivadinov R, Bosco A, Bragadin LM, Moretti R, Bonfigli L, Morassi P, Iona LG, Cazzato G (1999). Sexual dysfunction in multiple sderosis: a case-control study. 1. Frequency and comparison of groups. Multiple Sclerosis J.

[9] Zhang Y, Zhang Q, Shi Z, Chen H, Wang J, Yan C, Du Q, Qiu Y, Zhao Z, Zhou H (2020). Sexual dysfunction in patients with neuromyelitis optica spectrum disorder. J Neuroimmunol.

[10] Frequency of bladder, bowel and sexual dysfunction in NMO. J Neurol Neurosurg Psychiatry. 2014;85(10):e4.

[11] Wingerchuk DM, Banwell B, Bennett JL, Cabre P, Carroll W, Chitnis T, De Seze J, Fujihara K, Greenberg B, Jacob A (2015). International consensus diagnostic criteria for neuromyelitis optica spectrum disorders. Neurology.

[12] Anis TH, Gheit SA, Saied HS, Al kherbash SA (2011). Arabic translation of Female Sexual Function Index and validation in an Egyptian population. J Sex Med.

[13] Al-Musawi NmM (2001). Psychometric Properties of the Beck Depression Inventory-II With University Students in Bahrain. J Personality Assess.

[14] Al-Sobayel HI, Al-Hugail HA, AlSaif RM, Albawardi NM, Alnahdi AH, Daif AM, Al-Arfaj HF (2016). Validation of an Arabic version of Fatigue Severity Scale. Saudi Med J.

[15] Ansari NN, Naghdi S, Hasson S, Mousakhani A, Nouriyan A, Omidvar Z (2009). Inter-rater reliability of the Modified Modified Ashworth Scale as a clinical tool in measurements of post-stroke elbow flexor spasticity. NeuroRehabilitation.

[16] Kurtzke JF (1983). Rating neurologic impairment in multiple sclerosis: an expanded disability status scale (EDSS). Neurology.

[17] Pan J, Zhao P, Cai H, Su L, Wood K, Shi FD, Fu Y (2015). Hypoxemia, Sleep Disturbances, and Depression Correlated with Fatigue in Neuromyelitis Optica Spectrum Disorder. CNS Neurosci Ther.

[18] Seok JM, Choi M, Cho EB, Lee HL, Kim BJ, Lee KH, Song P, Joo EY, Min JH (2017). Fatigue in patients with neuromyelitis optica spectrum disorder and its impact on quality of life. PLoS One.

[19] Kef S, Bos H (2006). Is Love Blind? Sexual Behavior and Psychological Adjustment of Adolescents with Blindness. Sex Disability.

[20] Zhong S, Pinto JM, Wroblewski KE, McClintock MK (2018). Sensory Dysfunction and Sexuality in the U.S. Population of Older Adults. J Sex Med.

[21] Herz RS, Cahill ED (1997). Differential use of sensory information in sexual behavior as a function of gender. Human Nature.

[22] Bakır S, Penbegül N, Gün R, Yorgancilar E, Kiniş V, Özbay M, Atar M, Güneş M (2013). Relationship between hearing loss and sexual dysfunction. J Laryngol Otol.

[23] Levin R (2004). Smells and tastes–their putative influence on sexual activity in humans. Sex Relationship Ther.

[24] Hatfield RW. Touch and human sexuality. Human sexuality: An encyclopedia 1994:178–192.

[25] Ribeiro AM, Ferreira CHJ, Cristine Lemes Mateus-Vasconcelos E, Moroni RM, Brito LMO, Brito LGO (2014). Physical therapy in the management of pelvic floor muscles hypertonia in a woman with hereditary spastic paraplegia. Case Reports Obstetrics Gynecol.

[26] De Ridder D, Vermeulen C, De Smet E, Van Poppel H, Ketelaer P, Baert L (1998). Clinical assessment of pelvic floor dysfunction in multiple sclerosis: Urodynamic and neurological correlates. Neurourol Urodynamics.

[27] Bortolami A, Vanti C, Banchelli F, Guccione AA, Pillastrini P (2015). Relationship Between Female Pelvic Floor Dysfunction and Sexual Dysfunction: An Observational Study. J Sex Med.

